# Multiscale Analysis of Surface Topography for Engineering Applications in the Casting Industry

**DOI:** 10.3390/ma17215272

**Published:** 2024-10-30

**Authors:** Damian Gogolewski, Tomasz Kozior, Paweł Zmarzły

**Affiliations:** Faculty of Mechatronics and Mechanical Engineering, Kielce University of Technology, Al. Tysiąclecia Państwa Polskiego 7, 25-314 Kielce, Poland; tkozior@tu.kielce.pl (T.K.); pzmarzly@tu.kielce.pl (P.Z.)

**Keywords:** wavelet transformation, multiscale analysis, surface topography, 3D printing, casting industry

## Abstract

This paper presents the results of studies aimed at assessing the impact of the molding process on the variability of surface irregularities of casting models. This research was conducted using a selected multiscale method, i.e., wavelet transformation, in both discrete and continuous perspective. The test samples were made both based on traditional methods of manufacturing casting models, i.e., machining of aluminum and wood, as well as using three additive technologies. The impact of the forming process on the variability of the topography of the produced models was evaluated. This research comprehensively relates to the assessment of the applicability of additive technologies, which are increasingly used in various industrial areas, as well as the impact of the process on surface topography in relation to scale. The statistical assessment based on the ANOVA analysis demonstrated that it is possible to distinguish between the surfaces before and after a specific number of forming cycles. Studies have shown that the impact of the forming process is relatively small, mainly affecting the long-term irregularity components, and there are no functional dependencies in terms of the impact of the forming process on the variation in surface topography in relation to the manufacturing method or its parameters.

## 1. Introduction

The development of science and technology observed in recent years has created new opportunities for the potential use of modern solutions to modernize the technological processes of individual companies. Achieving the required functional characteristics, appropriate quality of the top layer, or dimensional compliance results in additive technologies being increasingly applied in specific areas of production. One of the key areas where their potential application could be significant is the casting industry [1,2,3,4]. During the molding process, casting models are largely exposed to bending. This process for polyamide-based materials has been presented in a previous study [5]. A large area of application for molds and casting models is the medical and dental industry, where numerous research papers have been published [6,7,8]. Fast production of the first sample and the ability to evaluate it and implement potential corrective measures leads to an increase in the competitiveness of the company and potential financial benefits. It shortens the process of implementing mass production and enhances the quality of the models produced. It is of considerable significance that a high level of production flexibility in the casting industry can be achieved specifically through the implementation of 3D printing technology.

In a previous publication [2], a comprehensive review of the possibilities of applying selected types of additive technologies in the casting industry for the production of casting molds is presented. They demonstrated that combining additive methods with traditional methods of manufacturing casting molds increases production flexibility and improves the work environment. The subject of ref. [9] pertains to the evaluation of the potential use of 3D printing technology in the creation of molds and casting models. The advantages and limitations of 3D printing technology in casting were demonstrated. In ref. [10], the impact of the technological parameters of FDM, SLS, and PJM technologies on the waviness of the surface of the casting models is presented. It has been demonstrated that the height of a single layer of building material significantly affects the values of waviness parameters. The studies presented in ref. [11] concern the evaluation of the potential use of Fused Deposition Modeling (FDM) additive technology for applying plate patterns to sand casting. The dimensional accuracy of the created models was assessed, and it was determined that the dimensions of the printed elements fell within the tolerance limits.

Analyzing the current state of knowledge, it should be noted that in the evaluation of additively manufactured surfaces, the classical approach based on parametric evaluation and Gaussian transformation seems insufficient [12,13]. The geometric complexity of the surface, with the formation of many defects such as overmelts, powder particles of various diameters that have not been fully sintered, the formation of additional randomly distributed morphological features, and other process errors reflected on the surface of the elements, mean that a parametric description based on statistical measures does not allow for a full picture of the surface complexity [14]. Consequently, the transfer of key information is used for diagnostic analysis of its functional, user characteristics. Therefore, in recent years, new multiscale methods have been developed that allow for a comprehensive assessment of such surfaces and the detection of features that are often not highlighted in the results of analysis using classical algorithms [15,16]. They allow for the characterization of properties, surface features in relation to scale, and the discovery of interactions between the functional properties of elements and the distribution of irregularities on their surface. Evaluating the topography of the surface in relation to scale potentially allows for the definition of surface characteristics, and subsequently also for the diagnosis of the production process. There are many types of algorithms that allow for the evaluation of 2D and 3D surface signals/profiles. They are used in many engineering fields for a more precise description of individual phenomena and for in-depth characterization of information [17,18,19,20,21]. In this regard, approaches based on both geometric methods [22], bandpass filters, structural functions, or hybrid methods are a combination of multiscale methods and classically used algorithms, including Fourier analysis [15].

One of the multiscale methods used for surface topography analysis is wavelet transformation. The multiplicity of mother wavelets with different characteristic properties determines the possibility of optimizing the filtration process in terms of identifying additional morphological features on the surface [23]. The potentially large capabilities in diagnosing surface irregularities and sensitivity to relatively small changes allow us to state that this is an effective tool for detailed, comprehensive analysis [24,25]. The use of a series of high- and low-pass filters allows for a multi-dimensional assessment of irregularity distributions, ranging from high-frequency changes to irregularities with a smoother character. However, it is crucial to consider the characteristics of individual waveforms and their potential impact on the results of filtration. There are numerous studies indicating the advantages and disadvantages of various types of transformations; hence, it seems purposeful to analyze wavelets from different groups. Individual wavelets differ not only in support width, which depends on the number of vanishing moments, and wavelet order, but also in variable symmetry, orthogonality, and variability of filters in decomposition and reconstruction. When analyzing non-periodic irregularities of surface topography, the use of the Morlet wavelet appears to be beneficial, which is characterized by non-orthogonality and can be characterized as a complex sinusoidal function enveloped by a Gaussian function centered at a specific frequency [26]. Analyzing the current state-of-the-art, selected wavelets can be applied for the evaluation of periodic, non-periodic, or chaos signals [27,28,29]. The wavelet approach enabling the assessment of individual irregularity distributions in both a discrete and continuous manner allows for the evaluation of individual surface features and the influence of the production process, post-processing, or any other components affecting the variability of the topography. This is particularly important for comparative surface analysis and the assessment of factors influencing the quality of the casting model in the molding process.

This article presents the results of experimental studies evaluating the impact of the molding process (one hundred molding cycles) on the variability of the surface topography of samples made with selected additive technologies, taking into account selected technological parameters. The assessment of surface topography was conducted using a selected multiscale method, wavelet transformation, in both discrete and continuous approaches, employing a series of mother wavelet types on 2D and 3D surfaces. Currently, there is a lack of research related to multiscale analysis of the impact of the forming process on the surface topography, particularly of casting models made with 3D printing. The conducted research fills the research gap and enhances the potential possibilities of applying additive technologies and modern multiscale methods in aspects of their use in the casting industry. This study provides information about the distribution of irregularities and morphological features on the surface of samples produced additively, as well as the influence of actual forming conditions on the variability of surface irregularities of casting models. The ability to distinguish between 2D and 3D surface profiles obtained before and after the forming process provides information on the impact of the forming process on surface topography variability and allows for a better understanding of the potential wear of casting models. Furthermore, the knowledge gained will allow for the selection of additive technology parameters to obtain casting models whose surfaces will be more resistant to wear as a result of the forming process.

## 2. Materials and Methods

**Figure 1:**
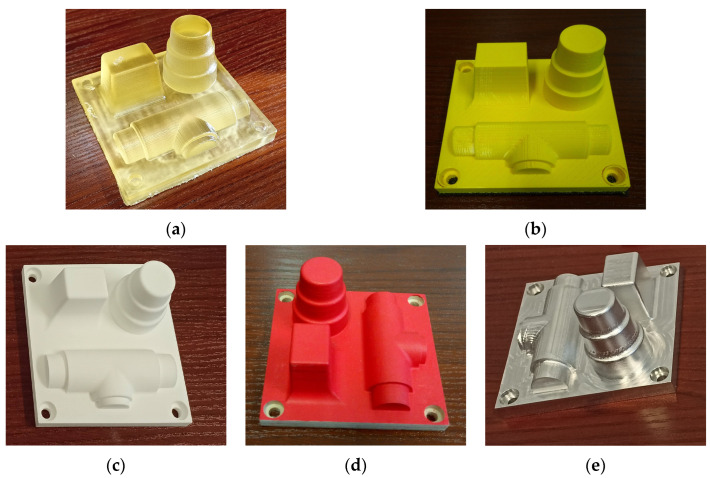
View of the evaluated samples: (**a**) PJM, (**b**) FDM, (**c**) SLS, (**d**) wood, (**e**) aluminum.

The test samples were fabricated employing three additive technologies: PJM, FDM, and SLS. For each technology, the intention was to assess the impact of the variability of selected technological parameters in terms of their effect on surface topography irregularities. In PJM technology, the Connex 350 printer (Stratasys, Eden Prairie, MN, USA) and FullCure 720 material (Stratasys, Eden Prairie, MN, USA) were used. The technological parameters analyzed were the thickness of the layer, which was selected as Lt = 0.016 mm and Lt = 0.032 mm, and the printing direction of 0° and 90° (Figure 1a). In the FDM technology (MEX—material extrusion, according to the new ISO/ASTM 52900 standard) [30], a Dimension 1200ES printer (Stratasys, Eden Prairie, MN, USA) and ABS P430 material (Stratasys, Eden Prairie, MN, USA) were used. The technological parameters were Lt = 0.254 mm or Lt = 0.33 mm, and the printing direction was either 0° or 90° (Figure 1b). In the case of the third technology (SLS, Figure 1c), a Formiga P100 printer (EOS GmbH, Krailling, Germany) and PA 2200 (EOS GmbH, Krailling, Germany) material (based on PA 12 polyamide) were used. The variables being analyzed included energy density—0.056 J/mm^2^ and 0.08 J/mm^2^, layer thickness Lt = 0.1 mm, Lt = 0.2 mm, as well as the printing direction—0° and 90°. The results of the studies on the variability of irregularity distributions before and after a specified number of forming cycles were compared with the distribution of surface irregularities for casting models manufactured using conventional methods from wood (Figure 1d) and aluminum (Figure 1e). The design of the test samples was created to represent typical characteristics of casting models, including the gradient and rounding radii, etc. The molding process was carried out under conditions similar to industrial conditions, i.e., manual molding was carried out using a pneumatic rammer.

**Figure 2:**
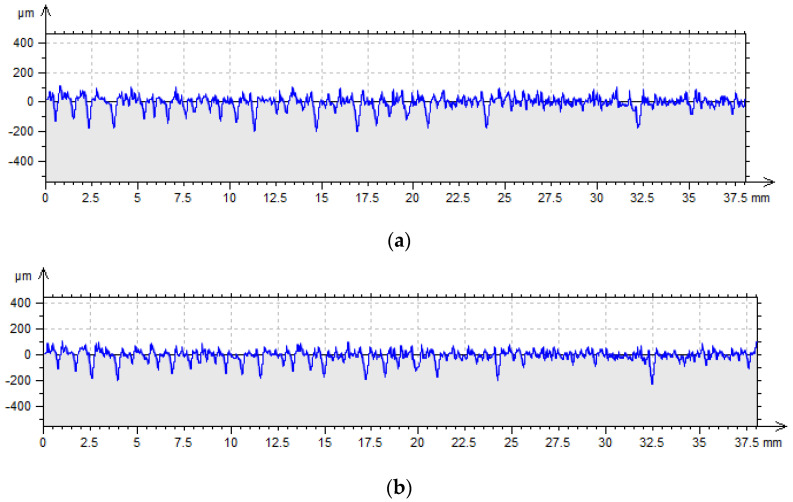
Example of surface profile: (**a**) before the forming process, (**b**) after the forming process (x axis—measurement length, y axis—irregularities height).

The topography measurements were conducted on a Form Talysurf PGI1200 stylus profilometer (Taylor Hobson, Leicester, UK), whereas the 3D surfaces were measured using an optical measuring instrument based on the coherence correlation interferometry principle—Talysurf CCI Lite (Taylor Hobson, Leicester, UK). Figure 2 shows a sample made using PJM technology at a print angle of 90° and a layer thickness Lt of 0.016 mm, including an example profile before (Figure 2a) and after the forming process (Figure 2b). Research using wavelet transformation was carried out on measured 2D profiles and 3D surfaces without conducting additional operations, such as filtering.

Considering the significant influence of the mother wavelet shape on the filtration results, this research was carried out using various types of mother wavelets with different characteristics. For the evaluation of distributions using a discrete approach, the following wavelets were selected: db2, db12, db20, coif2, coif5, sym2, sym8, bior1.5, bior5.5, and dmey. To comprehensively approach the problem, an analysis was also conducted based on the continuous wavelet transform using the Morlet wavelet. In a 2D approach, the wavelet is defined by Equation (1) [31], whereas in a 3D approach, it is defined by Equation (2) [32]. In these studies, the values *σ* = 1, *ε* = 1 *ω*_0_ = 6 were adopted.

(1) $$\Psi \left(x\right)=\pi^{-1/4}e^{i\omega_{0}t}e^{-\frac{x^{2}}{2}}$$

(2) $$\Psi \left(x,y\right)=e^{-\sigma^{2}\left({\left(x-\omega_{0}\right)}^{2}+\frac{{\left(\varepsilon y\right)}^{2}}{2}\right)}$$

One of the tools based on wavelet transformation, allowing for the assessment of the impact of the formation process on the variability of the topography of surfaces of elements produced by 3D printing, is wavelet coherence analysis, defined by relation (3):

(3) $$W_{xy}^{2}\left(a,b\right)=\frac{{\left|{\bar{W}}_{xy}\left(a,b\right)\right|}^{2}}{{\left|{\bar{W}}_{x}\left(a,b\right)\right|}^{2}{\left|{\bar{W}}_{y}\left(a,b\right)\right|}^{2}}$$

where *W_xy_*(*a*,*b*) is the coherence coefficient determined for two profiles marked as *x* and *y*; $\bar{W}$*_xy_*(*a*,*b*) is a smoothed cross spectrum $\bar{W}$*_x_*(*a*,*b*), and $\bar{W}$*_y_*(*a*,*b*) are smoothed wavelet spectra. The parameter accepts values in the range of 0 to 1, where the higher the value, the greater the coherence of both profiles.

Statistical assessment of the values obtained, including evaluation of the profiles, was carried out based on the *p*-value parameter, which was calculated using one-way ANOVA. The calculations were performed in MATLAB R2021a (MathWorks, Natick, MA, USA). It is accepted that a critical value sufficient for determining similarity is *p* > 0.05. Calculations were implemented for each level of decomposition and mother wavelet. Shapiro–Wilk tests were used to test for the normal distributions of residuals.

## 3. Results and Discussion

**Figure 3:**
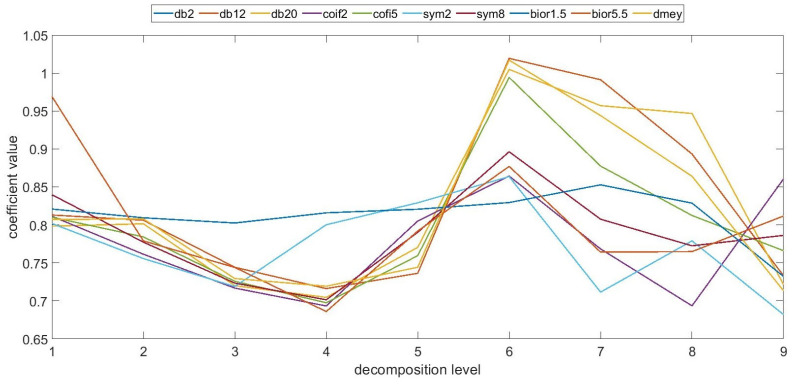
An example of the coefficient values determined for FDM technology—2D profile.

**Figure 4:**
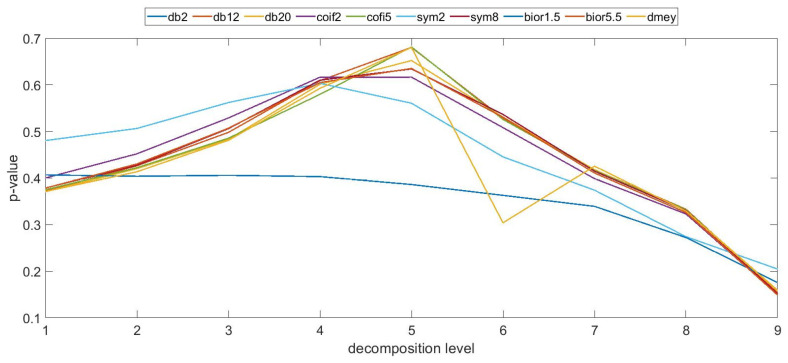
*p*-value as a function of mother wavelet and decomposition level, root mean square height parameter—FDM technology.

In these studies, a series of parameters related to surface topography were used, including the root mean square height, which is widely used in the assessment of the distributions parameter. This factor has been determined for individual profiles and surfaces, enabling a quantitative assessment of irregularities at various levels of discrete wavelet transformation. Figure 3, based on the authors’ research in a prior study [33], shows exemplary values of the coefficient determining the ratio of the root mean square height parameter calculated for the surface before and after the forming process. Based on the conducted analysis, it can be observed that the forming process affected the variability of the surface irregularities of the analyzed samples. Changes were observed for both high-frequency and low-frequency information, albeit with varying intensities. When comparing the examined manufacturing technologies, one can observe a varying intensity of the impact of the molding mass and the molding process on the surface topography distribution. The ANOVA statistical analysis demonstrated that it is feasible to distinguish between surfaces before and after a specific number of forming cycles for individual samples. However, this depends on the technological parameters and production methods. However, as exemplified by FDM technology, for which ref. [33] presented sample results of the molding process effect, in Figure 4, it can be seen that the individual profiles before and after the molding process are quite similar, especially at the initial levels of analysis, which present information about the high frequency of the profiles. Nevertheless, one can observe a characteristic trend of the statistical value obtained for the evaluated parameter. For higher values of the scale describing low-frequency information, the degree of profile similarity statistically decreases. It can be inferred that the molding process had an impact on the long-term components of the surface profile, which are potentially more sensitive to the effects of the molding process (the impact of the rammer and the molding mass on the casting model surface).

Based on the tests conducted, no statistically significant differences were found for most mother wavelets in terms of their impact on the filtration results and the ability to distinguish between 2D and 3D surface profiles. Significant differences were noted for the mother wavelet bior1.5 on most of the analyzed decomposition levels. The study conducted in terms of wavelet properties showed that although the wavelet transformation is a lossless analysis for this mother wavelet, the characteristics of the decomposition and reconstruction filters are different, which may affect the results of filtering and reconstruction. Similarly, for the dmey mother wavelet, there was an anomaly in the presented values with respect to the other wavelets. For the sixth level, a lower value of the coefficient was noted. For this level of decomposition, the scale of irregularities, including the similarity of the profiles before and after the molding process, was relatively small. It can be concluded that at this level, different types of information were filtered out for the profiles before and after the molding process, which may provide a diagnostic indication in terms of the evaluation of profiles and the impact of the process on topography variability. However, this is an unusual situation that occurred randomly for the indicated technology.

**Figure 5:**
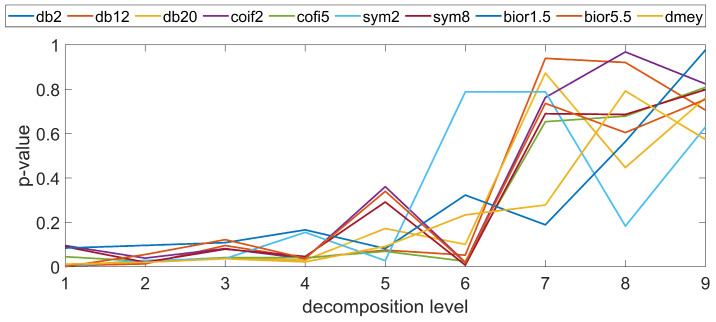
*p*-value as a function of mother wavelet and decomposition level, root mean square height parameter—PJM technology.

The studies conducted for the remaining additive technologies used, as well as for samples produced via conventional methods from wood and aluminum, did not show a similar trend in terms of the impact of the forming process on the surface topography variability for all technologies. It can be observed that for individual samples produced with specific technological parameters, the conducted wavelet analysis resulted in the detection of dependencies in individual frequency bands and scales, but functional dependencies do not occur. Additionally, individual cases appeared randomly depending on the manufacturing technology and applied parameters. The example results for PJM technology presented in Figure 5 do not converge to the statistical values presented in Figure 4. The value of the statistics at the initial stages is significantly lower compared to FDM technology; however, this trend reverses for low-frequency components. For the remaining assessed production methods, the obtained values are not correlated with the presented results; hence, it can be inferred that the forming process affects the models to varying degrees, depending on the type of technology and technological parameters or material, impacting more significantly on the low-frequency or high-frequency components of the surface irregularities.

**Figure 6:**
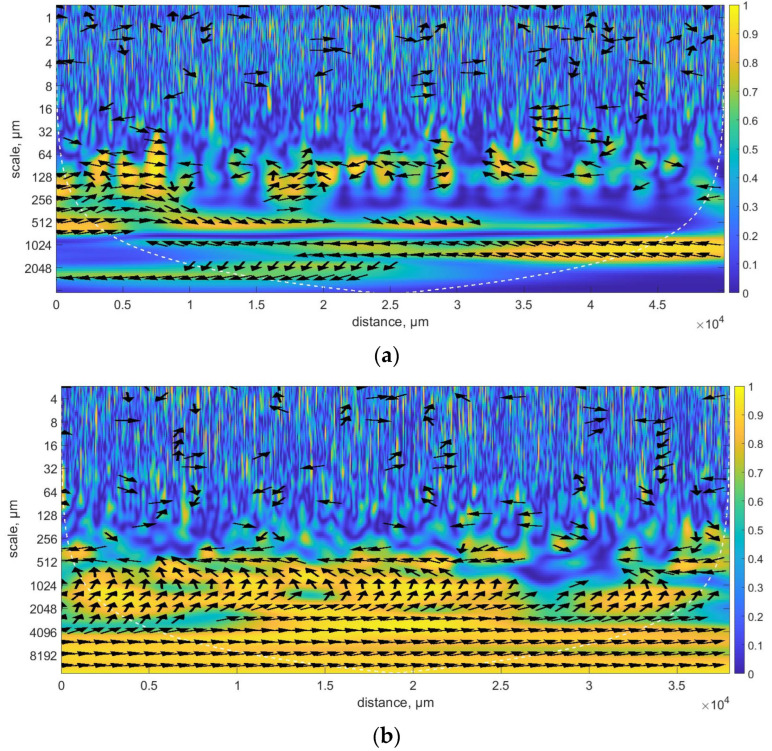
Wavelet coherent power spectrum between profiles before and after the forming process—example of (**a**) FDM, (**b**) SLS.

An analysis using wavelet transformation in a discrete approach also requires the evaluation of the correlation of filtration results at individual levels of wavelet decomposition. This research demonstrated that the obtained values did not show any similarities between the profiles. The aim was therefore to assess which areas potential similarities of profiles before and after the molding process could occur. Therefore, it was considered appropriate to conduct an analysis using continuous wavelet transformation. The analysis allowed us to determine the similarity both in terms of scale as well as the occurrence of corresponding areas. Research based on the assessment of the correlation coefficient value for the wavelet coherence analysis method was carried out using the Morlet wavelet. The comparative assessment of the profiles before and after the forming process using a continuous approach in relation to the scale is depicted in Figure 6, where the results of the analysis for an exemplary pair of profiles before and after the formation of a sample made using FDM and SLS additive technology are presented.

Analyzing the presented results, one can conclude that there are certain bands for which a high value of the wavelet coherent power spectrum was recorded. The values of the coefficient depicted in the above Figure 6a were determined for a sample made at a 0° building angle and a layer thickness of 0.254 mm, while in Figure 6b, the results are shown for a sample made at a 90° angle and a layer thickness of 0.016 mm. Analyzing the overall results for individual technologies and printing directions, analogous dependencies were noted. For scales presenting high-frequency information, individual coefficient values were defined in a way that did not allow for the identification of trends, patterns, or the discovery of functional relationships connecting technological process parameters and the distribution of surface irregularities. Local similarities were noted for individual samples, suggesting that the forming process significantly influenced changes in the distribution of surface irregularities. The noted similarities were concentrated in the range of larger scales, of the order of at least twice the thickness of the layer being built, depending on the technology. From this perspective, it is significant that even for surfaces made at a 0° angle, the correlation coefficient values mainly focused around the 0.512 mm scale, which was also identical for samples made at a 90° angle. Nevertheless, for samples made at a 0° angle, there were also similarities noted for a smaller scale value, but not along the entire length of the profiles. Analyzing the arrows depicted in the diagram, it can be concluded that for Figure 6a and the band corresponding to the scale of 0.512 mm, most of the arrows are oriented eastwards, which indicates the same phase relationship throughout the entire period for both profiles, with significant coherence and without any obvious dominant dependence. However, for larger scales, the tendency is the opposite, and the arrows are directed westward, which indicates a phase opposition. For the coefficients presented in Figure 6b, it was noted that the arrows are primarily oriented in the northern direction, suggesting that both profiles on a given scale are similar. However, the irregularities are shifted by one-quarter of the cycle and scale size.

## 4. Conclusions

This research focuses on the assessment of the potential use of selected additive technologies for the construction of casting models, as well as evaluating the impact of the forming process on the variability of the distribution of irregularities in the produced models. The purpose of a comprehensive evaluation is to compare the research results obtained for the selected additive technologies with the results obtained for samples made via machining from wood and aluminum. The analysis of surface topography (2D and 3D) before and after the molding process was conducted using wavelet transformation.

1. Studies have shown that the impact of the forming process is relatively small. Changes have been noted for both high-frequency information and low-frequency information, with varying intensity. Changes occurred in the irregularity schedule caused both by the influence of the forming mass and the forming process, and ANOVA statistical tests, among others, showed that conducting one-hundred forming cycles only slightly affected the variability of the surface topography. These changes were noted for the high-frequency components of the surface irregularities; however, local correlations between the profile before and after the molding process were observed, as well as for subsequent levels of decomposition describing low-frequency information, for which the degree of similarity of the profiles statistically decreased. It can be stated that the forming process had a greater influence on the long-term components of the surface profile.
2. Assessment of profiles based on continuous wavelet transformation allowed us to conclude that for most of the evaluated samples, high coefficient values were primarily concentrated around the scale of 0.512 mm. Utilizing a continuous approach, it was noted that for low frequencies, there was a phase shift, which could have been reflected in the values obtained in the case of discrete analysis.
3. It should be noted that no significant influence of individual mother wavelets was observed in terms of surface discrimination. The selected forms of wavelets, which were characterized by various properties and filtration capabilities of the profiles, did not allow us to obtain statistically different data for individual levels of wavelet decomposition. The exception was the bior1.5 wavelet, for which significantly different results were obtained, and the dmey wavelet, for which there was an anomaly in the presented values with respect to the other wavelets at the selected level. This may provide a diagnostic indication in terms of the evaluation of the profiles and the impact of the process on the topography variability.
4. The studies conducted did not show convergent trends in terms of the impact of the forming process on the variability of the surface topography with respect to the manufacturing method. It can be observed that for individual samples produced using specific technological parameters, the conducted wavelet analysis resulted in the detection of dependencies in individual frequency bands and scales, but functional dependencies did not occur, and individual cases appeared randomly depending on the manufacturing technology and applied parameters. This research enhances the practical applicability of new surface irregularity assessment techniques developed within the framework of Metrology 4.0, and it increases the practical use of additive technologies in relation to their application in the casting industry.

Further research will focus on evaluating the potential application of wavelet analysis for rapid diagnosis of both surface topography and shape error assessment, both as an alternative algorithm and in hybrid models that are a combination of wavelet transformation and traditional analysis methods. This will allow for a comprehensive analysis of the quality of casting models and their resistance to the molding process.

## Data Availability

The raw data supporting the conclusions of this article will be made available by the authors on request.

## Abbreviations

*biorNr.Nd*	Mother wavelet biorthogonal, Nr defines wavelet order of scaling functions used for reconstruction; Nd defines the wavelet order of the functions used for decomposition
*coifN*	Mother wavelet Coiflet, *N* defines the number of vanishing moments
*dbN*	Mother wavelet Daubechies, *N* defines the number of vanishing moments
*dmey*	Discrete Meyer wavelet
*FDM*	Fused Deposition Modelling
Lt	Layer thickness
PJM	PolyJet Matrix
SLS	Selective Laser Sintering
*symN*	Mother wavelet Symlet, *N* defines the number of vanishing moments
*ψ*(*x*)	Mother wavelet
